# Structure and ultrastructure of nuptial and extranuptial nectaries explain secretion changes throughout flower lifetime and allow for multiple ecological interactions

**DOI:** 10.1093/aobpla/plaf037

**Published:** 2025-07-04

**Authors:** Hannelise Balduino, Priscila Tunes, Massimo Nepi, Elza Guimarães, Silvia Rodrigues Machado

**Affiliations:** Postgraduate Program in Plant Biology, São Paulo State University, R. Prof. Dr. Antônio Celso Wagner Zanin 250, 18618-689 Botucatu, SP, Brazil; Department of Biodiversity and Biostatistics, Laboratory of Ecology and Evolution of Plant-Animal Interactions, São Paulo State University, R. Prof. Dr. Antônio Celso Wagner Zanin 250, 18618-689 Botucatu, SP, Brazil; Department of Life Sciences, University of Siena, Via Mattioli 4, 53110 Siena, Italy; National Biodiversity Future Centre (NBFC), Piazza Marina 61, 90133 Palermo, Italy; Department of Biodiversity and Biostatistics, Laboratory of Ecology and Evolution of Plant-Animal Interactions, São Paulo State University, R. Prof. Dr. Antônio Celso Wagner Zanin 250, 18618-689 Botucatu, SP, Brazil; Department of Biodiversity and Biostatistics, Laboratory of Ecology and Evolution of Plant-Animal Interactions, São Paulo State University, R. Prof. Dr. Antônio Celso Wagner Zanin 250, 18618-689 Botucatu, SP, Brazil; Microscopy Center, São Paulo State University, R. Prof. Dr. Antônio Celso Wagner Zanin 250, 18618-689 Botucatu, SP, Brazil; Natural History and Conservation

**Keywords:** ant, bees, extranuptial nectary, mutualism, nuptial nectary, ultrastructure

## Abstract

Nectaries are specialized nectar-producing structures. Nectar traits affect animal behaviour and ecological and evolutionary processes, such as pollination and biotic defence. Previously, we found that there are differences in the characteristics of nuptial nectar (NN) and extranuptial nectar (ENN) and in the types of animals that visit each nectary in *Amphilophium mansoanum* (Bignoniaceae) flowers. We now hypothesize that nectar traits reflect the anatomical, histochemical, and subcellular characteristics of each nectary type. Using routine light microscopy, scanning electron microscopy, and transmission electron microscopy methods, we studied NN and ENN, respectively in young flower buds, pre-anthesis buds, and first- and second-day flowers. NN was a prominent annular disk; whereas, ENN was a concave, patelliform trichome. Only ENN contained alkaloids, while both nectaries contained starch grains, lipid droplets, proteins, terpenes, and phenolic compounds. Both nectaries showed subcellular organization consistent with hydrophilic and lipophilic secretion, the latter being predominant in second-day flowers. In NN, the subnectary parenchyma had phloem and amyloplasts until pre-anthesis. Starch grains decreased and tracheary elements were seen in newly opened flowers. ENN are not vascularized, with vascular bundles from the calyx approaching the base of the nectary. Starch grains were scarce and very small in the ENN secretory head cells. Fibrillary proteins were found only in NN and periplastidial reticulum was observed only in ENN. In NN, nectar secretion begins shortly before anthesis, being released through the raised stomata and the reticulate cuticle. In ENN, secretion extends from the young flower bud stage to senescent flowers, accumulating in small subcuticular spaces and being continuously released through the intact cuticle. Temporal differences in NN and ENN production, as well as in the structural characteristics and nectar release mechanisms between NN and ENN, may explain the differences in nectar characteristics of *A. mansoanum*.

## Teaser

We found in a previous study (published in AoB Plants https://doi.org/10.1093/aobpla/plac067) that there are differences in the characteristics of nuptial and extranuptial nectar and in the types of animals that visit each nectary in Amphilophium mansoanum (Bignoniaceae) flowers. Thus, in the present paper, we hypothesize that the specificities of nectar from nuptial and extranuptial nectaries of A. mansoanum reflect underlying subcellular specializations. We found that in nuptial nectaries, nectar secretion begins shortly before anthesis, being released through raised stomata and the reticulate cuticle. Whereas in extranuptial nectaries, secretion extends from the young flower bud stage to senescent flowers, accumulating in small subcuticular spaces and being continuously released through the intact cuticle. Moreover, temporal differences in nuptial and extranuptial nectar production, as well as in the structural characteristics and nectar release mechanisms between nuptial and extranuptial nectaries, may explain the differences in nectar characteristics of A. mansoanum.

## Introduction

Nectar, a sugar-rich secretion, is a highly energetic and ready-to-consume primary resource for several animals (Nicolson 2007, 2022, Nepi et al. 2018). When these animals forage among different plant species, they come across nectar with different volume, chemical profiles (including sugars, amino acids, proteins, lipids, organic acids, and secondary compounds), and concentrations (Calixto et al. 2021, Barberis et al. 2023, Zhou et al. 2024). Due to the effects of these traits on animal’s foraging behaviour and movement, nectar is an important mediator of mutualisms between plants and animals, including pollination and indirect defence against herbivores (Nicolson 2007, 2022, Heil 2015, McPeek et al. 2021, Mogensen et al. 2024). When plants and animals exert selective pressures on each other, these interactions can either increase or decrease reproductive and survival success, thereby affecting ecological and evolutionary processes (Nepi 2017, Nepi et al. 2018, Suissa et al. 2024).

Nectaries, i.e. structures specialized in nectar production, show a wide range of sizes, morphology, anatomy, and locations (Erbar 2014 and references therein), which may influence nectar traits (Petanidou et al. 2000, Nepi 2007). Based on their topography, they are further categorized as floral or extrafloral nectaries (Caspary 1848). From a functional perspective, nectaries can be classified as nuptial, when associated with pollination, or extranuptial, when associated with defence (Delpino 1873).

Considering that nectaries have evolved independently across angiosperms (Weber and Keeler 2013, Phukela et al. 2020), including different lineages of Bignonieae (Bignoniaceae) (Nogueira et al. 2013), and the cost of nectar production for plants (Southwick 1984, Pyke 1991), it is expected that mechanisms of nectar synthesis and release would be variable among plant organs (Nepi 2007, Chatt et al. 2021) and flower developmental stages (Nepi 2007, Guimarães et al. 2016). Besides that, traits such as vascularization, may severely affect nectar traits (Frey-Wyssling 1955, Galetto 1995). Therefore, studying nectary micromorphology, anatomy, and ultrastructure is essential to understand the relationships between plants, visiting animals, and the environment (Kostryco and Chwil 2022, Almeida et al. 2023).

Members of the Bignoniaceae family, including *Amphilophium mansoanum* (DC.) L.G. Lohmann [sin. *Distictella mansoana* (Vahl) Urb.], commonly have two nectary types in the same species, nuptial and extranuptial, which are usually morphologically different (Galetto 1995, Balduino et al. 2023) and have distinct evolutionary origins (Lohmann and Taylor 2014). Nuptial nectaries (NN) are typically annular disks at the ovary base (Thomas and Dave 1992, Galetto 1995, 2009, Rivera 2000a, Bernardello 2007, Guimarães et al. 2016, 2018), composed of a unistratified epidermis, nectary and subnectary parenchyma supplied with phloem (*sensu* Nepi 2007). Their secretion begins before anthesis, so that nectar is already available when a flower opens (Guimarães et al. 2016, 2018). Nectar-secreting cells contain abundant mitochondria, rough endoplasmic reticulum, Golgi bodies with numerous vesicles, and amyloplasts, which degrade as anthesis progresses (Guimarães et al. 2016, 2018, Machado et al. 2017). Secretion may shift from hydrophilic to lipophilic during anthesis, which is distinguished by an increase in plastoglobuli, and is released through modified stomata, cuticular pores, or microchannels (Guimarães et al. 2016, 2018, Machado et al. 2016). In contrast, extranuptial nectaries (ENN) are glandular trichomes that vary in shape, size, number, and location (Seibert 1948, Rivera 2000b, Nogueira et al. 2013, Fróes et al. 2015, Miranda et al. 2024). These trichomes are composed of a stalk and a multicellular secretory head, with cells showing abundant mitochondria, endoplasmic reticulum, leucoplasts, few dictyosomes, and wall ingrowths with plasmodesmata between the stalk and the secretory head cells (Gama et al. 2016). Different types of nectar-secreting trichomes (putative nectaries) may occur adjacently on the same perianth whorl (Elias and Gelband 1976, Galetto 1995, Nogueira et al. 2013, Fróes et al. 2015).

In a previous study with *A*. *mansoanum*, two types of nectaries were found within a given flower, nuptial (annular disk) and extranuptial (patelliform glands near the calyx margins), which nectar differs in composition, volume, concentration, and secretion dynamics (Balduino et al. 2023). Furthermore, since NN are visited by medium and large bees, while ENN are visited mainly by ants. Nectar from both nectary types differed regarding chemical composition, being NN sucrose-dominated and ENN hexose-rich, the first had a more variable amino acid profile than the latter and had a theophylline-like alkaloid, which was exclusive from NN (Balduino et al. 2023).

While this previous study focused on the ecological and chemical aspects of nectar, in this research we aim, to expand upon those findings by investigating the anatomy, ultrastructure and histochemistry of the NN and ENN that co-reside in *A*. *mansoanum* flowers from a developmental perspective. Studying the structure and functioning of the different nectaries within a given flower expands our understanding on the relationships between nectaries and their secretions, which mediate plant–animal interactions. We hypothesize that the specificities of nectar from NN and ENN of *A*. *mansoanum* (shown in Balduino et al. 2023) reflect underlying subcellular specializations. Beyond that, nectaries that coexist within the same flower are subjected to the same environmental and physiological conditions. Therefore, by comparing the structural mechanisms that govern the nectar secretion process in distinct nectaries and their respective functioning, we can gain deeper insights into how nectary features relate to their ecological functions.

## Materials and methods

### Plant material and study site

**Figure 1.**
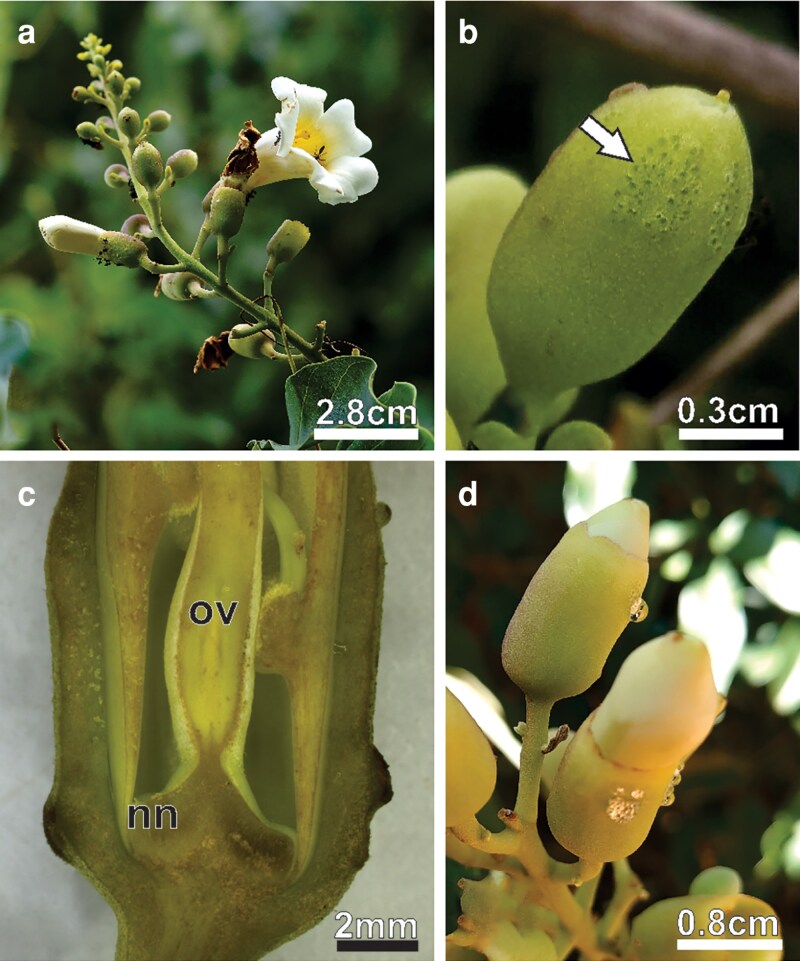
Inflorescence and flowers of *A. mansoanum* (Bignoniaceae). (a) Inflorescence with flowers in different developmental stages. Note ants on the calyx margin in the pre-anthesis bud. (b) Clustered prominent pores arranged in longitudinal bands at the distal region of the young bud. (c) Pre-anthesis bud sectioned longitudinally in the median plane showing a nectary at the base of the ovary and nectariferous chamber. (d) ENN droplets at the calyx margin of the young and pre-anthesis buds.

*Amphilophium mansoanum* is a liana native to Brazil but not endemic, exhibiting a broad geographic distribution across the country, particularly on the edges of tropical forests and savannas (Lohmann and Taylor 2014). The zygomorphic, pentamerous flowers are arranged in racemose panicles, although simple racemes may occasionally occur (Pool 2009) (Fig. 1a). The dome-shaped calyx is coriaceous with a cluster of glands near its margins (Fig. 1b). The corolla is infundibuliform, bent at ca. 90° above the base, coriaceous, with five lobes, imbricate, white and internally yellow (Fig. 1a). The androecium has four stamens and included anthers. The ovary is sessile, and the nectary disk is annular (Fig. 1c). Drops of viscous secretion (Fig. 1d) occur on the calyx surface from young floral buds to open flowers.

We sampled flowers (*n* = 3 flowers per stage, four plants) at four different stages of development: young buds (10–20 mm), pre-anthesis buds (40–50 mm), freshly opened flowers (first-day flowers) and flowers around 24 h from opening (second-day flowers). The samples of the NN and ENN were collected in the morning hours from natural populations of *A*. *mansoanum* at the edge of cerrado vegetation in the Midwest of São Paulo State, Brazil (22° 53′ 56″ S and 48° 29′ 68″ W; 966 m), from November 2021 to February 2023. A voucher specimen was deposited at the BOTU herbarium (Águas de Santa Bárbara, São Paulo, Brazil) under number 41575. The collection was made by T.C. Monteiro and H.K. Balduino.

### Structural, histochemical, and ultrastructural studies

For general histological characterization, samples were fixed in 2.5% glutaraldehyde in 0.025 M phosphate buffer (Feder and O’Brien 1968) in field conditions and maintained overnight at 4°C in the laboratory. Specimens were dehydrated through graded series of ethanol solutions (60%–100%), and embedded in glycol methacrylate historesin (Leica Microsystems, Nussloch/Heidelberg, Germany). Thin sections (4–6 μm) were obtained using a semiautomatic microtome (Leica RM2245), placed on microscopic glass slides, stained with 1% toluidine blue in 1% aqueous sodium tetraborate solution (O’Brien et al. 1964) and mounted in synthetic resin (Entellan, Merck KgaA, Darmstadt, Germany). The results of the light microscopic examinations were documented with a photomicroscope (Leica DMR) equipped with a digital camera (Leica DFC 500).

For histochemical studies, fresh NN and ENN samples were hand-cut using a razor blades and subjected to the following treatments: Alcian blue for polysaccharides and acid polysaccharides (da Silva Figueiredo et al. 2007); Lugol’s reagent for alkaloids and starch grains (Johansen 1940); ferric chloride for phenolic compounds (Johansen 1940); mercuric bromophenol blue for proteins (Mazia et al. 1953); NADI reagent (α-naphthol + dimethyl-*p*-phenylenediamine) for essential oils and oleo-resins (David and Carde 1964); ruthenium red for pectin (Jensen 1962); and Sudan IV for total lipids (Johansen 1940). When required, standard control sections were prepared at the same time. All observations and documentation were performed using a light microscope (Leica DMR) with a digital camera (Leica DFC 500).

For surface examination, portions of the flowers were cut with razor blades in the median longitudinal and transversal planes. The samples were fixed in 2.5% glutaraldehyde (0.1 mol l^−1^ phosphate buffer, pH 7.2), dehydrated in an aqueous ethanolic series, critical-point dried (Bal-Tec CPD 030), mounted on a metallic support with adhesive, and sputtered coated with gold (10 nm) (Bal-Tec SCD 050) following Bozzola and Russel (1992). The material was then examined with a scanning electron microscope (SEM Quanta 200, Fei Company, FEI, Gräfelfing, Germany) at 20 kV.

For cellular ultrastructure examination, the fragments were fixed in 2.5% glutaraldehyde (0.1 M sodium phosphate buffer pH 7.3, overnight at 4°C) and post-fixed with 1% osmium tetroxide (OsO_4_) in the same buffer for 2 h at room temperature (Hayat 1989). After washing in distilled water, the material was dehydrated in aqueous acetone and embedded in Araldite resin (Araldite 502, Electron Microscopy Sciences, Hatfield, USA). Semi-thin sections (0.5 μm) were obtained using an ultramicrotome (Leica Reichert) and stained with 0.05% toluidine blue (O’Brien et al. 1964). Ultrathin sections (60 nm) were stained with 2% uranyl acetate solution for 15 min and lead citrate for 15 min (Reynolds 1963) and observed with a transmission electron microscope (TEM Tecnai Spirit, FEI Company, Germany) at 60 kV.

We also employed the zinc iodide–osmium tetroxide (ZIO) method for endomembrane impregnation (Reinecke and Walther 1978). After primary fixation, the samples were incubated in a solution containing Zn, I, Tris-aminomethane, and 1.0% osmium tetroxide (Reinecke and Walther 1978), and processed conventionally for TEM.

## Results

### Nuptial nectary structure, ultrastructure, and secretion

**Figure 2.**
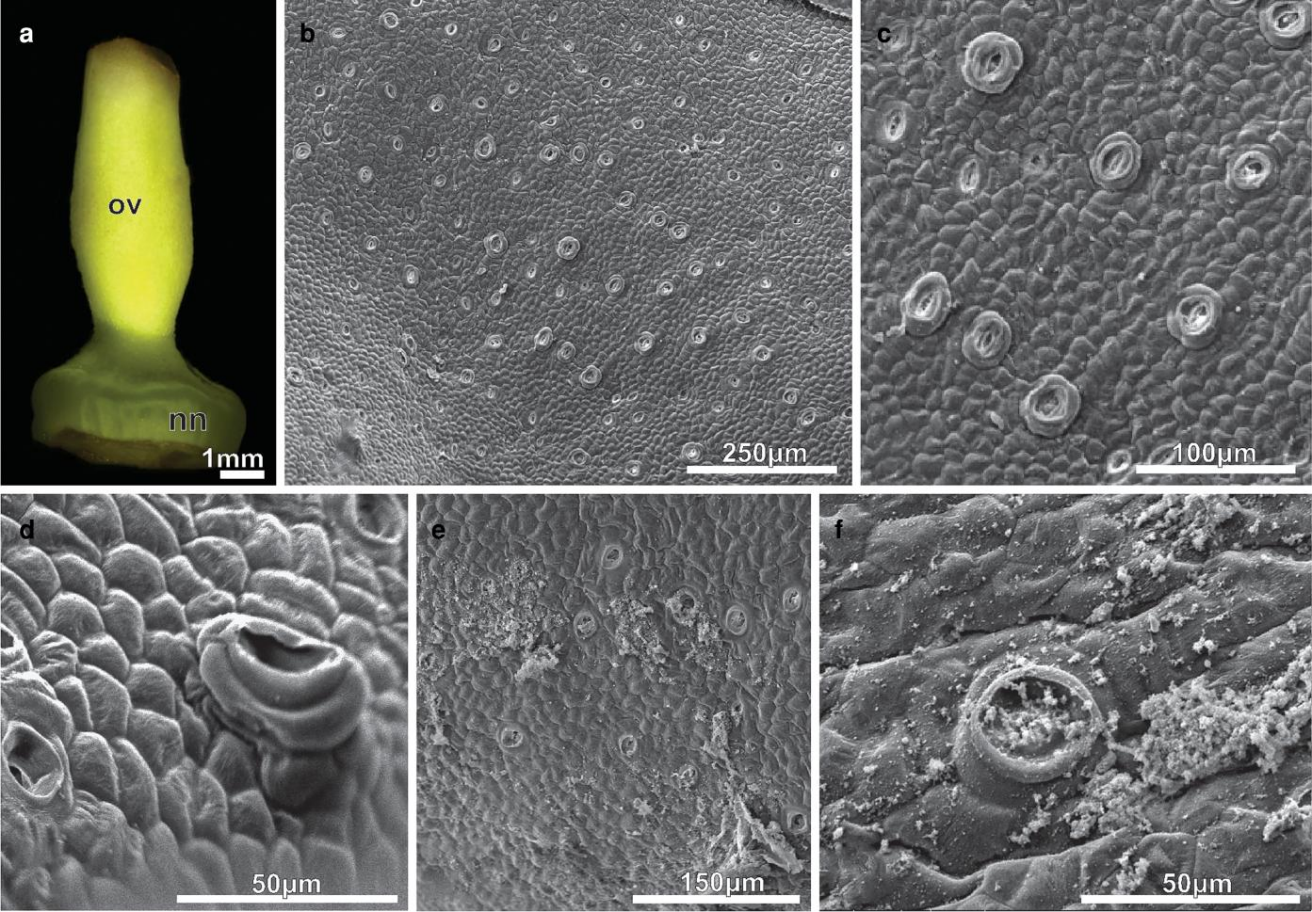
Micrographs of the NN of *A. mansoanum* (Bignoniaceae). (a) Appearance of the nectary disk in pre-anthesis buds seen under a stereomicroscope. (b–f) SEM micrographs. (b) Stomata irregularly distributed on the nectary disk surface. (c) Detail showing ordinary stomata together with prominent large stomata with wide pores. Note the guard cells with cuticular ledges. (d) Giant raised stoma. (e) First day flower; nectary disk covered with flocculant secretion residues. (f) Raised stoma with wide circular pore filled with secretion residues.

The NN is an annular disk located below the ovary (Figs 1c and  2a). The expanded base of the stamen filaments creates a constriction that surrounds the nectary and forms a nectar chamber (Fig. 1c), where the secreted nectar builds up. Nectar secretion begins in pre-anthesis floral buds. At this developmental stage, nectary surface had common epidermal cells with different sizes and shapes, with anticlinal walls that were undulating to different degrees and external periclinal walls sticking out (Fig. 2b and c). Numerous stomata also with different shapes and sizes were distributed across the surface of the nectary (Fig. 2b and c). The curvature of the external periclinal wall varied from flat to slightly convex to rounded (Fig. 2c). These included ordinary stomata with a long narrow opening and an outer stomatal rim, as well as stomata that were sunken or raised (Fig. 2b and c). Giant and raised stomata with a large opening and stomatal border, elevated by their subsidiary cells, were commonly observed (Fig. 2d). First-day flowers showed flocculent secretion residues covering the nectary surface (Fig. 2e). At this point, the giant raised stomata had a bigger round opening that was full of secretion residues (Fig. 2f).

**Figure 3.**
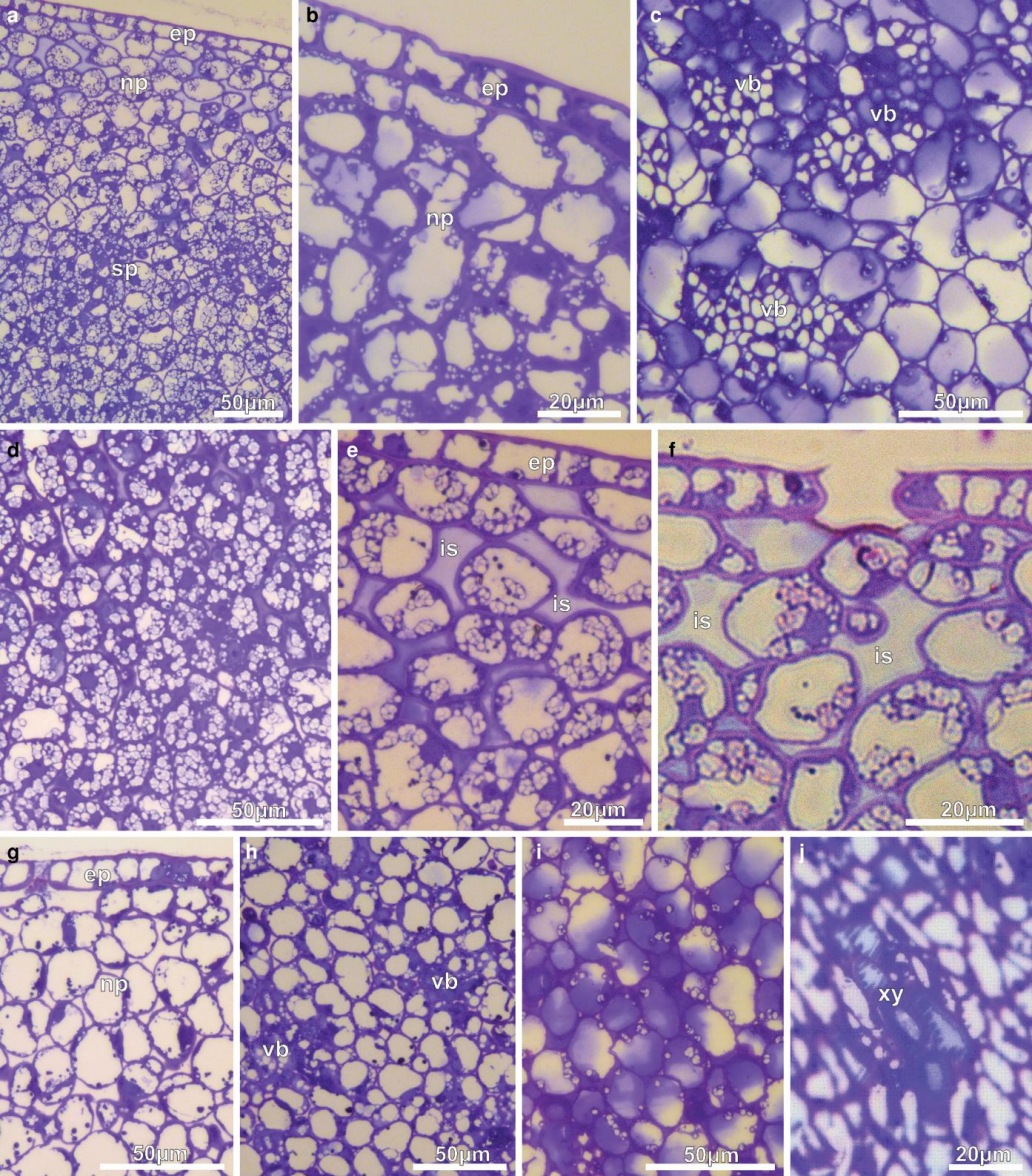
Light micrographs of cross-sections of the NN of *A. mansoanum* (Bignoniaceae). (a–f) Pre-anthesis buds. (a) General view of the nectary with epidermis, nectary parenchyma underneath the epidermis, and subnectary parenchyma. (b) Detail showing scarce starch grains in the parenchyma. (c) Vascular bundles in the subnectary parenchyma. Note predominance of phloem. (d) Abundance of starch grains in the subnectary parenchyma. (e) Intercellular spaces filled with secretions in the nectary parenchyma. (f) Stoma with a wide opening. (g–j) First-day flowers. (g) Nectary parenchyma showing rounded cells with large vacuole and dense accumulations. (h) Subnectary parenchyma with vacuolated cells. (i) Parenchyma cells with phenolic compounds and sparse voluminous starch grains. (j) Tracheary elements in the subnectary parenchyma. Key to figures: ep, epidermis; ie intercellular space; np, nectary parenchyma; sp, subnectary parenchyma; vb, vascular bundles; xy, xylem.

Cross-sections of the NN of pre-anthesis buds showed three different regions under a light microscope (Fig. 3a). They featured a single-layered epidermis, a nectary parenchyma situated beneath the epidermis, and a large subnectary parenchyma that was densely vascularized by vascular bundles originating from the flower receptacle (Fig. 3a). At this stage, a clear separation of the nectary and subnectary parenchyma was evident (Fig. 3a): the nectary parenchyma was made up of conspicuous cells with an ovoid to isodiametric shape that were arranged in three to five non-vascularized parenchymal layers (Fig. 3b); the subnectary parenchyma was made up of ovoid cells that were arranged in several layers with proliferating vascular bundles, mostly phloem (Fig. 3c). On the first day of anthesis, flowers had an increase in voluminous starch grains in the subnectary parenchyma cells (Fig. 3d), while in the nectary parenchyma, the intercellular spaces developed and were filled with secretions (Fig. 3e). Stomata with large open pores are evident at this stage (Fig. 3f). On the second day of anthesis, the epidermal cells and parenchyma cells displayed dense accumulations (Fig. 3g and h), while the starch grains were either absent or scarce (Fig. 3i). At this stage, parenchyma cells contained homogenous content (Fig. 3i), probably phenolic compounds, and tracheary elements in the subnectary parenchyma (Fig. 3j).

**Figure 4.**
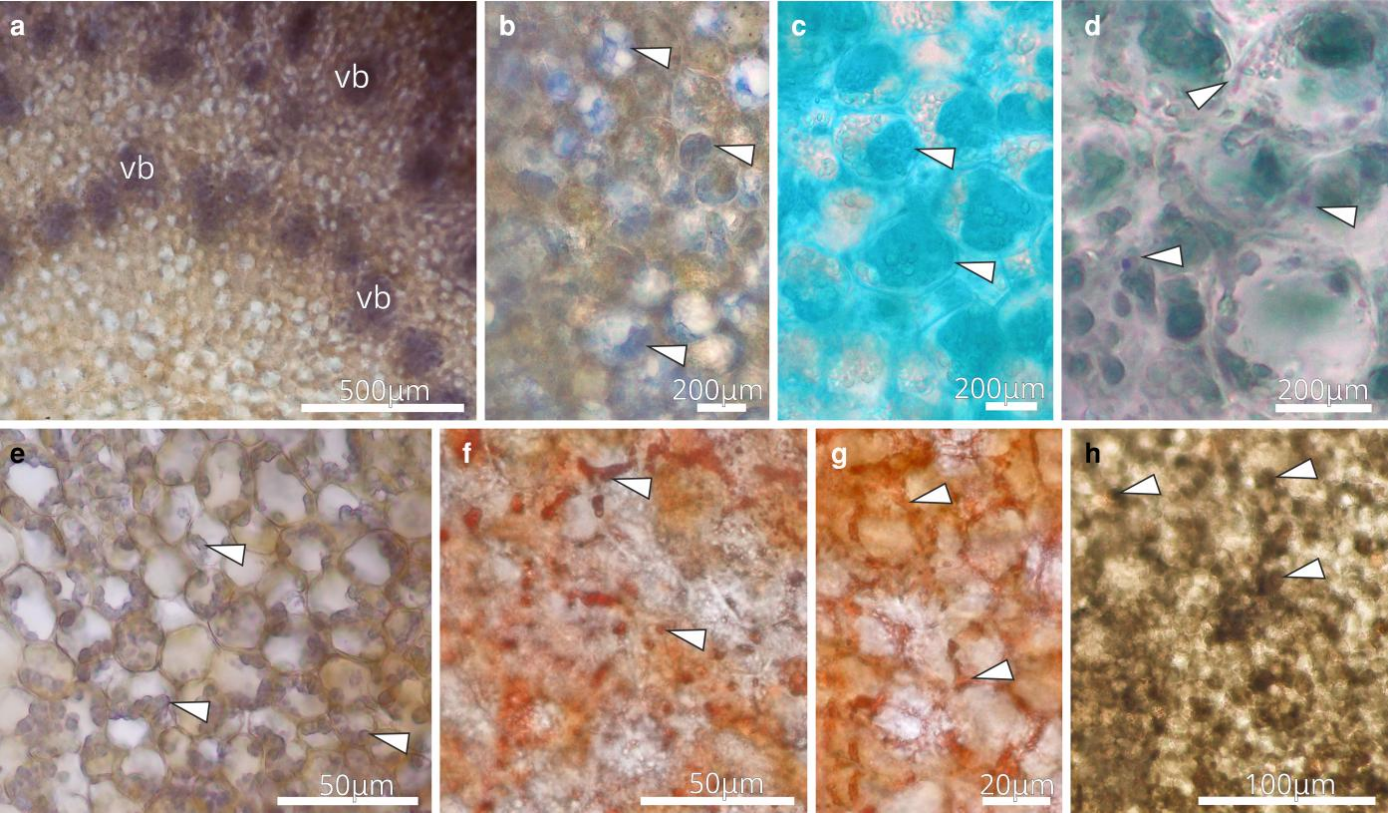
Histochemical characterization of the NN of *A. mansoanum* (Bignoniaceae); cross-sections. (a and b) Positive reaction to test with mercury blue bromophenol reagent, evidencing the presence of basic proteins in the vascular bundles (vb), and in the parenchyma cells (arrowhead). (c) Positive reaction to test with Alcian blue, evidencing the presence of acid polysaccharides. (d) Positive reaction to the test with NADI reagent (arrowhead), evidencing the presence of essential oils (blue staining) and oils-resin (pink staining). (e) Positive reaction to test with Lugol reagent (arrowhead), evidencing the presence of starch grains. (f and g) Positive reaction to test with Sudan IV (arrowhead), evidencing the presence of lipids. (h) Positive reaction to test with ferric chloride reagent (arrowhead), evidencing the presence of phenolic compounds.

The histochemical characterization of the disk tissues of first-day flowers revealed positive reaction for hydrophilic and lipophilic substances (Fig. 4; Table 1). Tests with mercury blue bromophenol detected basic proteins stained in blue in the subnectary parenchyma cells, mainly in the vascular bundles (Fig. 4a), and in nectary parenchyma cells (Fig. 4b). Alcian blue highlighted acid polysaccharides substances (Fig. 4c). Tests with NADI reagent also showed a positive result, developing both a pink colouration (characteristic of oil-resins) and a blue colouration (characteristic of essential oils) in the parenchyma cells (Fig. 4d). Tests with Lugol revealed accumulation of big starch grains in all disk tissues, but more numerous in the subnectary parenchyma (Fig. 4e). Tests with Sudan IV stained in red lipid droplets (Fig. 4f) and accumulations of lipid material in the intercellular spaces (Fig. 4g). With ferric chloride, all the nectary cells were stained brown (Fig. 4h), indicating the presence of phenolic substances. Tests with ruthenium red stained mainly cell walls (Fig. 4i).

**Table 1.** Sites of histochemical reaction in NN and ENN of *A. mansoanum* flowers.

Stage	Nectary type	Reagents and target compounds							
		Ferric chloride (phenolic substances)	Lugol (starch grains)	Lugol (alkaloids)	NADI (terpenes)	Ruthenium red (pectin)	Sudan (lipids)	Alcian blue (acid polysaccharides)	Mercuric bromophenol blue (proteins)
Young bud	NN	snp (ic)	snp (ic)	snp (ic)	NA	ep (na) np (cw) snp (cw)	snp (ic)	NA	NA
	ENN	NA	ft (ic) st (ic) sh (ic)	NA	NA	sh (ic)	st (cw) sh (cu)	NA	NA
Pre-anthesis bud	NN	ep (ic) np (ic) snp (ic)	ep snp (ic)	ep (ic)	ep (ic; ex) np (ic) snp (ic)	ep (cu; ic; cw) np (is; cw; ic) snp (cw; vb)	ep (cu; ic) np (ic) snp (ic)	NA	NA
	ENN	sh (ic) ft (ic)	ft (ic)	ft (ic)	NA	ft (cw; ic), st (ic) sh (cw; ic)	ft (ic) st (cw; ic) sh (ic; cu; scu)	NA	NA
First-day flower	NN	ep (ic) np (ic) snp (ic)	ep (ic) np (ic) snp (ic)	np (ic) snp (ic)	ep (ex) np (ic) snp (ic; is)	ep (cw) np (cw) snp (cw)	ep (na) np (na) snp (is; ic)	ep (cw; ic) np (cw; ic) snp (cw; ic)	ep (ic) np (ic) snp (ic; vb)
	ENN	ft (ic)	ft (ic) sh (ic)	ft (ic) st (ic) sh (ic)	sh (ic)	ft (cw; is; ic) st (ic) sh (cw; ic)	st (cw) sh (cu)	sh (cw; scu) ft (is; cw)	sh (ic)
Second-day flower	NN	np (ic) snp (ic)	ep (ic) np (ic) snp (ic)	snp (ic)	ep (ic) np (ic) snp (ic)	ep (cw) np (cw; ic) snp (cw; vb; ic)	ep (cu; ic) np (ic) snp (ic)	NA	NA
	ENN	ft (ic) sh (ic)	sh (ic) ft (ic)	ft (ic)	NA	sh (ic) ft (ic; cw)	ft (ic) st (cw) sh (cu; ic)	NA	NA

These results are shown in Figs 4 and 8. NA, not applicable; inconclusive results; NN, nuptial nectary; ENN, extranuptial nectary; ep, epidermis; np, nectary parenchyma; snp, subnectary parenchyma; ft, foot cells; st, stalk cells; sh, secretory head; cw, cell wall; is, intercellular space; ic, intracellular; ex, extracuticular; cu, cuticle; scu, subcuticular space; vb, vascular bundles.

**Figure 5.**
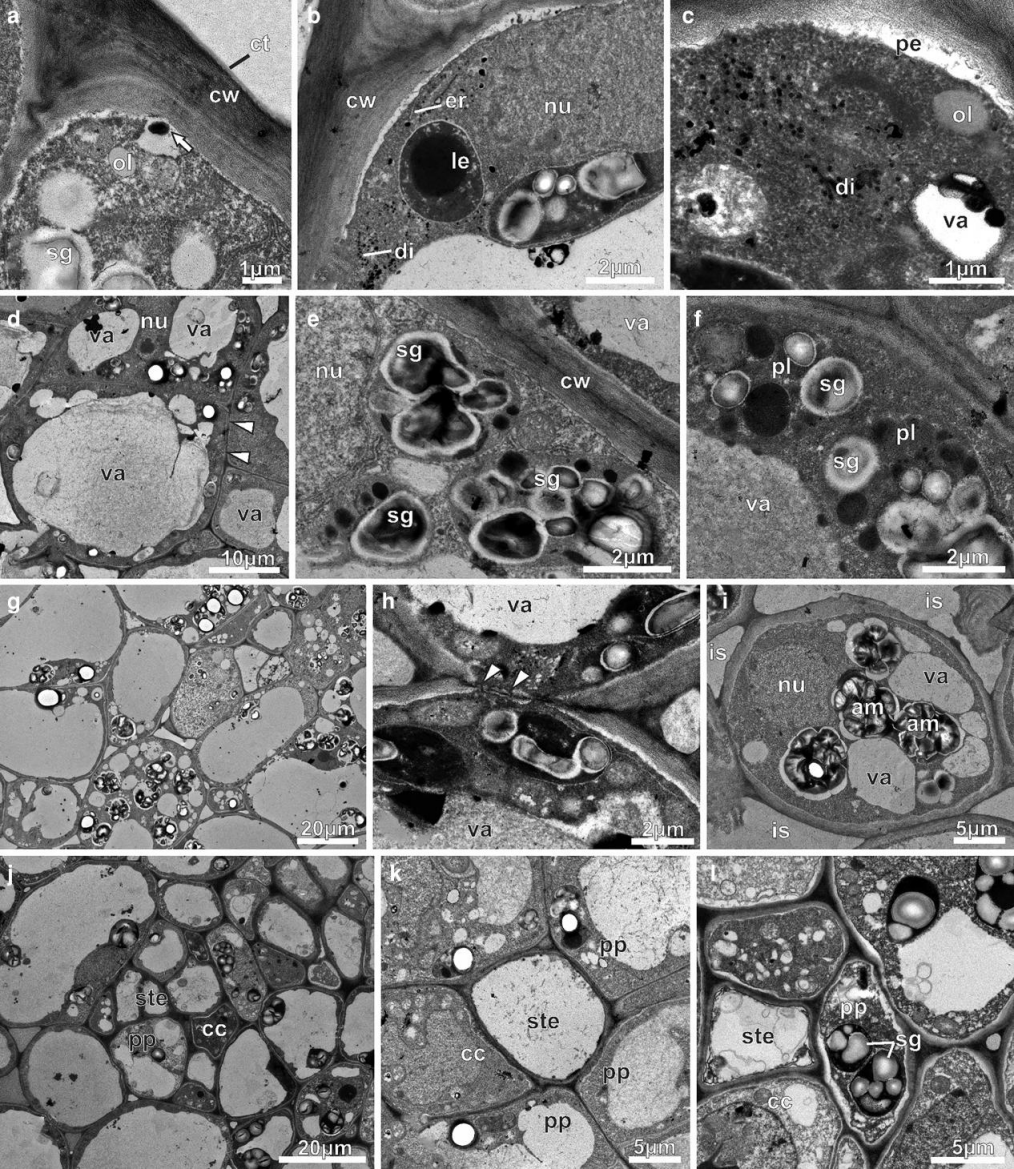
Transmission electron micrographs of the NN of *A. mansoanum* in pre-anthesis buds. (a–c) Epidermal cells. (a) Part of an epidermal cell with thick outer periclinal wall and thin cuticle, abundant cytoplasm, endoplasmic reticulum, starch grains and oil drops. Note the osmiophilic body in the periplasmic space (arrow). (b) Endoplasmic reticulum profiles at the peripheral cytoplasm, dictyosomes and vesicles marked by ZIO method, rounded leucoplast with a large osmiophilic inclusion, and ellipsoid amyloplast. Note electron dense granules in the periplasmic space and attached to the tonoplast. (c) Abundant vesicles from dictyosomes filled with dense content marked by ZIO method, and dense material inside the vacuole. Note the lipid drop at the peripheral cytoplasm. (d–f) Nectary parenchyma. (d) Parenchyma cells with very thin walls (arrowhead), nucleus and large vacuoles filled with flocculant materials. (e) Starch grains with different sizes and degradation signals. (f) Plastids with starch grains and osmiophilic globules. Note osmophilic bodies attached to the tonoplast. (g–i) Subnectary parenchyma. (g) Phloem and vacuolated parenchyma cells with big amyloplasts. (h) Plasmodesmata in the transversal walls connecting parenchyma cells (arrow heads). (i) Parenchyma cells with conspicuous nucleus, abundant cytoplasm, big amyloplasts and vacuoles with flocculent materials. Note the intercellular spaces with fibrillar materials. (j–l) Phloem in the subnectary parenchyma. (j) Several phloem cells in the subnectary parenchyma. (k) Sieve tube elements of large calibre. (l) Phloem cells with voluminous amyloplasts with intact starch grains. Key to figures: am, amyloplast; cc, companion cell; ct, cuticle; cw, cell wall; is, intercellular space; le, leucoplast; nu, nucleus; ol, oil drop; pe, periplasmic space; pp, phloem parenchyma; pl, plastid; sg, starch grain; sp, subnectary parenchyma; ste, sieve tube element; va, vacuole.

Under TEM, NN epidermal cells of the pre-anthesis buds displayed thick external periclinal walls covered by a thin cuticle (Fig. 5a). There were a lot of free ribosomes, mitochondria, plastids (Fig. 5b and c), and rough endoplasmic reticulum with cisternae and vesicles filled with dense content, which was marked by ZIO (Fig. 5c). Amyloplast containing starch grains and leucoplasts with dense matrix devoid of inner membranes and containing a large osmiophilic inclusion occurred side by side in the epidermal cell (Fig. 5b). The cytosol displayed oil drops (Fig. 5a and c). The vacuoles contained osmiophilic inclusions and flocculent materials (Fig. 5c). Accumulations of similar materials also occurred within the periplasmic space (Fig. 5a–c). In the nectary parenchyma (Fig. 5d–f), the cells exhibited compact arrangement, thin walls, large nuclei, and vacuoles of varied sizes (Fig. 5d), along with amyloplasts exhibiting different sizes and signs of degradation (Fig. 5e). These cells regularly displayed leucoplasts containing small starch grains and osmiophilic globules (terpenes/oils/phenols) (Fig. 5f). Inside the vacuoles, flocculent contents and membrane debris were observed (Fig. 5d and f). In the subnectary parenchyma, the cells were larger and contained numerous amyloplasts (Fig. 5g–i), as well as prominent nucleus (Fig. 5i). The vacuoles varied in size and contained flocculent materials (Fig. 5h and i). The intercellular spaces were filled with flocculant materials (Fig. 5i). Plasmodesmata were seen in the regions of contact between parenchyma cells (Fig. 5h). At this stage, the phloem was the main vascular tissue in the subnectary parenchyma (Fig. 5j), with sieve tube elements displaying a wide calibre (Fig. 5k), and parenchyma cells showing amyloplasts with voluminous starch grains (Fig. 5l).

**Figure 6.**
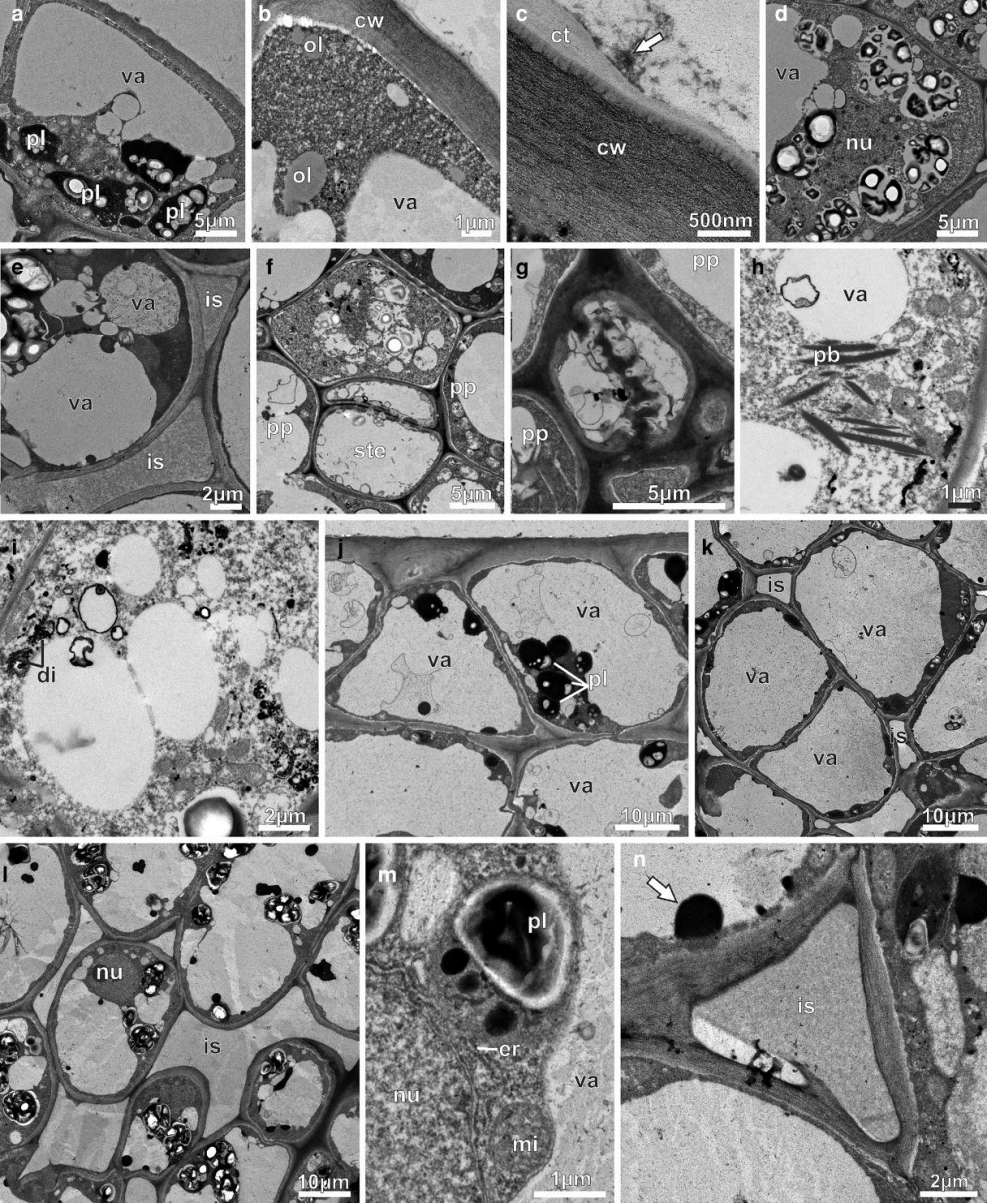
Transmission electron micrographs of the NN of *A. mansoanum* (Bignoniaceae). (a–i) First-day flowers; (j–n) second-day flowers. (a–c) Epidermal cells. (a) Large vacuole in the apical pole and clustered dense plastids with small starch grains in the basal pole. (b) Granulated cytoplasm with scattered oil drops, and vacuoles with flocculant material. Note the periplasmic space along the outer cell wall. (c) Secretion residues (arrow) on the cuticle. Note microchannels projecting from the cell wall to the cuticle. (d) Nectary parenchyma cell with collapsed amyloplasts and broken starch grains. (e) Subnectary parenchyma with vacuoles full of fibrillar or flocculant materials, membrane debris, and osmiophilic inclusions. Note larger intercellular spaces filled with fibrillar material. (f–i) Phloem features in the subnectary parenchyma. (f) Vacuolated parenchyma cell containing broken starch grains. (g) Sieve plate plugged with amorphous deposits, typically callose. (h) Parenchyma cell with spindle-shaped protein bodies scattered in the cytoplasm. (i) Parenchyma cell showing dictyosomes and vesicles marked by ZIO reaction. (j) Epidermal cells with reduced cytoplasm and big vacuoles with fibrillar inclusion and membrane debris. Note denser clustered plastids with tiny starch grains. (k) Nectary parenchyma showing vacuolated cells with reduced cytoplasm, and intercellular spaces. (l) Subnectary parenchyma with wider intercellular spaces; cells showing nucleus, reduced cytoplasm and big amyloplasts. Note dark inclusion in the vacuole. (m) Detail showing part of the nucleus, endoplasmic reticulum element, mitochondria, amyloplast containing one starch grain and osmiophilic bodies, and vacuole. (n) Detail of the subnectary parenchyma showing intercellular space filled with fibrillar material. Note dark inclusion in the vacuole (arrow). Key to figures: ct, cuticle; cw, cell wall; di, dictyosome; er, endoplasmic reticulum; ie, intercellular space; mi, mitochondria; nu, nucleus; ol, oil drop; pb, protein bodies; pp, phloem parenchyma; pl, plastid; ste, sieve tube element; va, vacuole.

The NN of first-day flowers contained epidermal cells characterized by large vacuoles at the distal pole (Fig. 6a) and densely granulated cytoplasm (Fig. 6b). Amyloplasts were localized at the base of the epidermal cells and were characterized by very electron-dense stroma and broken-down starch grains (Fig. 6a). Lipid droplets occurred dispersed in the cytoplasm and near the plasma membrane (Fig. 6b). The cuticle featured a large cuticular layer with a network of electron-dense ramifications that does not extend to the cuticle itself (Fig. 6c). This ultrastructural organization of the cuticle suggests a pattern resembling microchannels, through which secretion reaches the surface. Secretion residues were observed on the cuticle (Fig. 6c). At this stage, the nectary (Fig. 6d) and subnectary parenchyma (Fig. 6e) cells showed increased vacuolization and a higher number of amyloplasts, which consist of starch grains being broken down. Small vacuoles were either juxtaposed or fused with larger vacuoles (Fig. 6d and e), which contained fibrillary or flocculated materials, membrane debris, and osmiophilic inclusions (Fig. 6e). Intercellular spaces were wider and filled with flocculant material (Fig. 6e). In the phloem parenchyma cells, signs of degradation were noted in the amyloplasts (Fig. 6f). Additionally, we observed sieve tube elements with callose-plugged sieve plates (Fig. 6f and g). In the cytoplasm of phloem parenchyma cells, bundles of fibrillar protein were abundant, especially near the vacuoles (Fig. 6h). These cells had globular mitochondria, endoplasmic reticulum segments, dictyosomes, and ZIO marker-improved vesicles view (Fig. 6i).

In the NN of second-day flowers, cytoplasm was reduced to a thin parietal layer, and a central vacuole had been formed in epidermal (Fig. 6j), nectary parenchyma (Fig. 6k), and subnectary parenchyma cells (Fig. 6l). Amyloplasts (Fig. 6j) exhibited residual starch grains, dense stroma and large osmophilic inclusions (like terpenes/phenols). Subnectary parenchyma cells showed a big nucleus, larger vacuoles, and amyloplasts that were full of osmophilic substances (e.g. phenols and terpenes) and broken-down starch grains (Fig. 6l and m). These cells also exhibited segments of endoplasmic reticulum and mitochondria (Fig. 6m). Intercellular spaces were wider (Fig. 6l) and filled with flocculent material (Fig. 6n). Osmiophilic bodies were observed attached to the tonoplast (Fig. 6n).

### Extranuptial nectary structure, ultrastructure, and secretion

**Figure 7.**
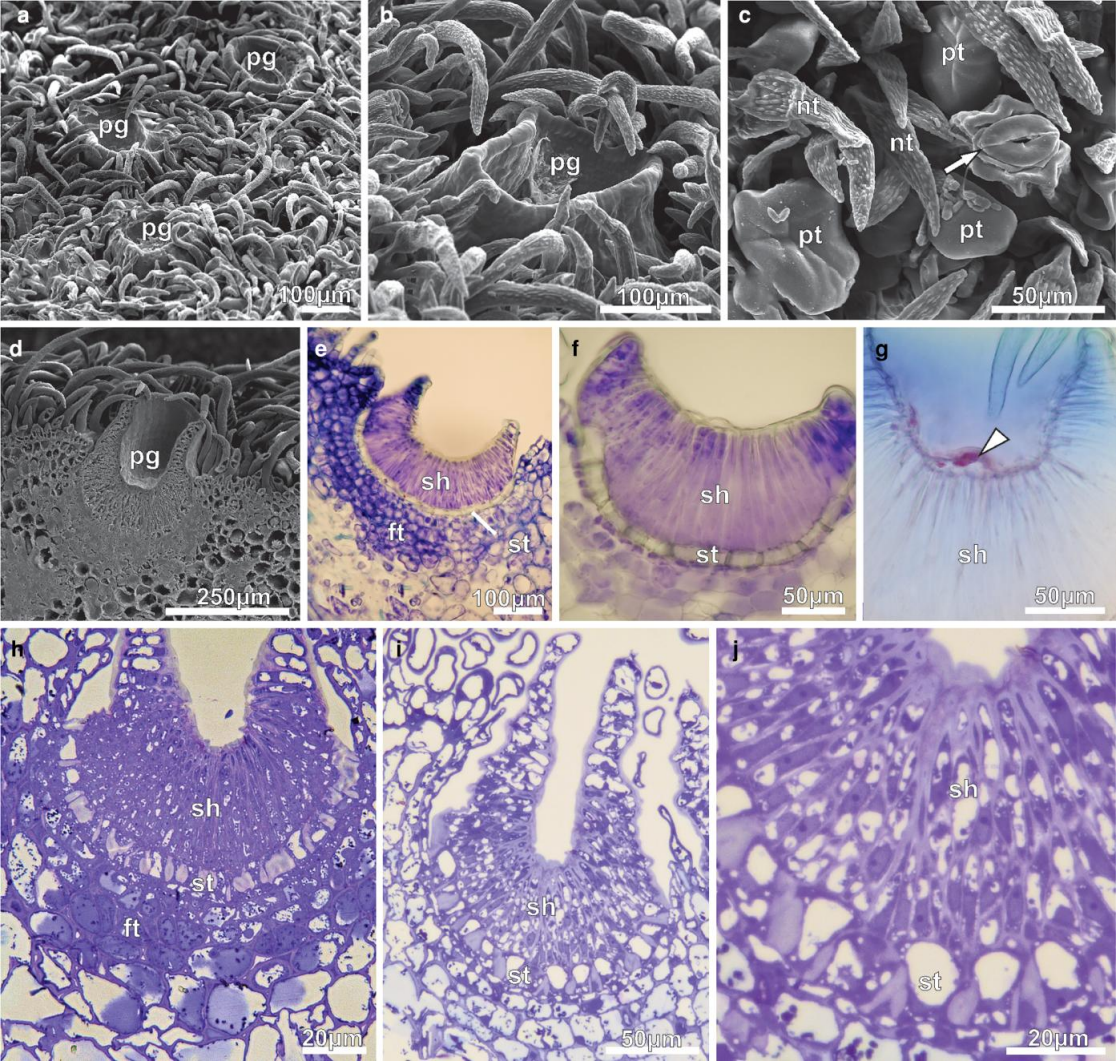
ENN of *A. mansoanum* (Bignoniaceae). (a–d) Scanning electron micrographs of the calyx surface. (a) Clustered concave glands at the surface of young flower buds surrounded by trichomes. (b) Gland with extended margin that projects above the surface of the calyx forming a wide central cavity. (c) Non-glandular trichomes, peltate glandular trichome, and raised stomata (arrow). (d) Gland sectioned longitudinally showing the concave portion located above the calyx surface and the portion immersed in the calyx mesophyll. (e–j) Light micrographs of the transversal section, stained with toluidine blue, showing glands at different developmental stages: (e–g) 6 µm thick; (h–j) 60 nm thick. (e and f) Young bud. (e) Gland formed by secretory head with undeveloped margin, uniseriate stalk, and parenchyma foot. (f) Detail showing head cells covered with a thick cuticle, and stalk cells with thick lateral walls. (g) Small subcuticular space in the central region of the secretory head with secretion accumulation (arrow). (h) Pre-anthesis bud; head secretory cells and foot cells with strongly stained dense cytoplasm. (i) First-day flowers; gland with expanded margin perpendicular to the surface of the calyx. Note that all gland cells have less dense content and more developed vacuoles. (j) Detail showing ticker cuticle, head cells with thick walls, prominent nucleus and vacuoles with different sizes. Note the thick-walled stalk cells and dense granulations in the foot cells. Key to figures: pg, patelliform-gland; pt, peltate glandular trichome; nt, non-glandular trichome; sh, secretory head; st, stalk; ft, foot.

**Figure 8.**
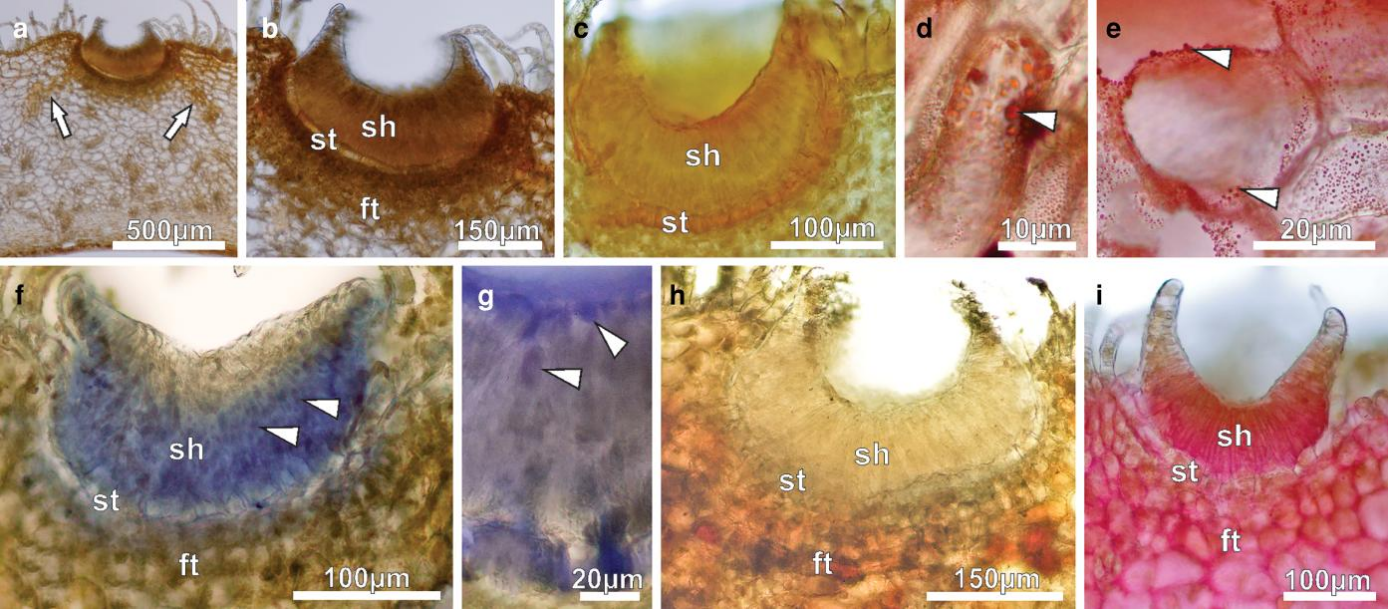
Histochemical characterization of the ENN of *A. mansoanum* (Bignoniaceae); cross-sections. (a and b) Positive reaction to starch grains and to alkaloids with Lugol. (a) Note the vascular bundles in the calyx mesophyll (arrowhead) ending at the foot of the gland. (b) Positive reaction more evident in the foot cells underlying the gland stalk and in the upper region of the secretory head. (c–e) Positive reaction to test with Sudan IV. (c) Presence of lipids in the lateral walls of the stalk cells and cuticle. (d) Lipid droplets (arrowhead) in the head cells. (e) Lipid droplets (arrowhead) in the foot cells. (f) Positive reaction to test with mercury blue bromophenol reagent, evidencing the presence of basic proteins in the head cells. (g) Positive reaction to test with NADI reagent (arrowhead), evidencing the presence of oils-resin (pink staining) in the head cells. (h) Positive reaction to test with ferric chloride reagent, evidencing the presence of alkaloids (stained reddish brown) in the foot cells. (i) Positive reaction to ruthenium red test, evidencing the presence of pectin in the cell walls of the central region of the secretory head and in the foot of the gland. Key to figures: ft, foot; sh, secretory head; st, stalk.

Under SEM, the secretion-producing areas on the calyx exhibited patelliform-concave glands surrounded by simple, uniseriate, and pluricellular non-glandular trichomes with pointy apical cells and warty ornamentations, as well as peltate glandular trichomes featuring a flat head and raised stomata (Fig. 7a–c). The extended margins of the patelliform glands protruded above the calyx surface (Fig. 7b and d). Transversal sections through the gland revealed that the concave portion was aligned with the epidermis, while the foot was immersed in the calyx mesophyll (Fig. 7d). The glands of young buds (Fig. 7e and f) did not possess fully developed edges, but displayed three distinct areas: (i) a concave, multicellular secretory head composed of very long, narrow, thin-walled cells covered with a thick cuticle; (ii) a short stalk that extends for the length of the secretory head; and (iii) a foot, or base, comprised of numerous tangentially expanded cells. A single layer of flattened, rectangular cells with thick anticlinal walls constitutes the stalk, connecting the secretory head to the nectary foot (Fig. 7e and f). The foot of the gland is not vascularized and consisted of three to five layers of cells with varied sizes and shapes, which contents are strongly stained with toluidine blue (Fig. 7e and f). Vascular bundles from the calyx mesophyll circumscribe the gland foot, but they do not extend through it (Fig. 8a).

In young buds, these glands are already secreting material despite the edges not being fully developed yet. The exudate was stored beneath the cuticle in the centre of their concave head (Fig. 7g). As differentiation progressed, the edge of the concave gland widened, and all gland cells, except for the stalk cells, became denser and more toluidine blue-stained (Fig. 7h). Progressively, the edges become more extended and the vacuoles in the head and foot cells become more developed (Fig. 7i) on first-day flowers. Additionally, the head secretory cells exhibit thicker walls. They possess a conspicuous nucleus with well-organized nucleolus, less cytoplasm, and several small vacuoles (Fig. 7j). The thick-walled cells in the gland stalk contained large a vacuole and reduced cytoplasm (Fig. 7j).

Histochemical analysis of mature ENN in first-day flowers revealed positive reaction for hydrophilic and lipophilic substances (Fig. 8; Table 1). With ferric chloride, phenolic substances were stained brown and were detected in all nectary cells (Fig. 8a), with a higher concentration in the gland’s apex (Fig. 8b). After Sudan IV tests, the thick cuticle and lateral cell walls of the stalk were better delimited (Fig. 8c). These tests also revealed that head secretory cells (Fig. 8d) and foot cells (Fig. 8e) contained abundant lipid droplets. Basic proteins were abundant in the head cells (Fig. 8f). Oil-resins, typically stained in pink with NADI reagent, were also found in the head cells (Fig. 8e). With Lugol’s reagent, small amounts of starch grains were detected in the head cells, whereas the contents of the foot cells acquired a reddish–brown colour (Fig. 8f), indicating the presence of alkaloids. Ruthenium red treatment evinced an abundance of pectin in the thick cell walls of the gland foot and of the central region of the secretory head (Fig. 8i).

**Figure 9.**
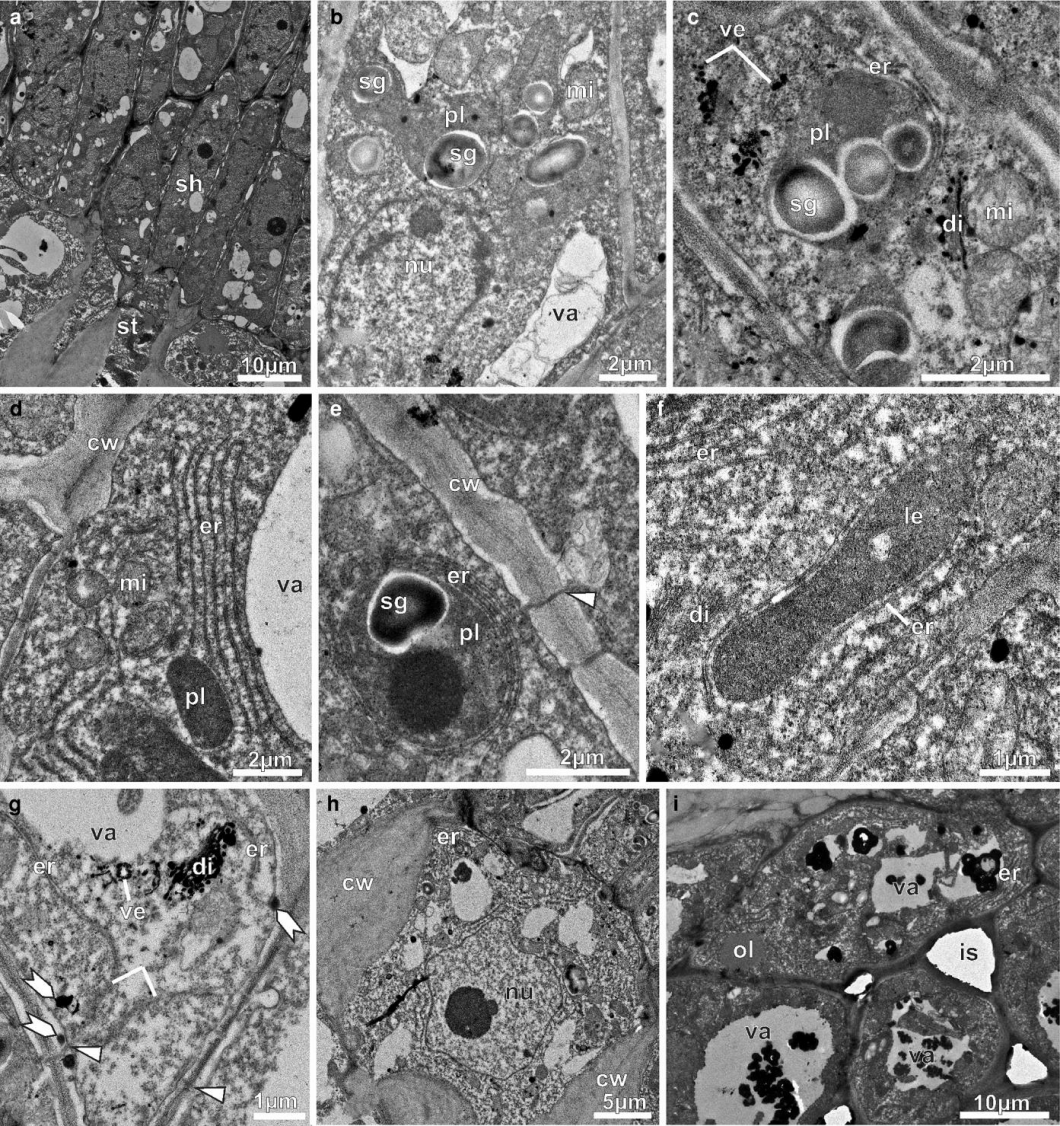
Transmission electron micrographs of the ENN of *A. mansoanum* (Bignoniaceae). (a–c) Glands in young buds; (d–i) glands in pre-anthesis buds. (a) Head cells with spherical and organized nucleolus, dense cytoplasm, and poorly developed vacuoles. (b) Amyloplasts with lots of starch grains, mitochondria, and small vacuole. (c) Amyloplasts, mitochondria, and dictyosomes with adjacent vesicles containing electron-dense material, marked by ZIO. (d) Mitochondria and extensive endoplasmic reticulum profiles. (e) Plastid with residual starch grains and a big osmiophilic inclusion. Note the endoplasmic reticulum around the plastid, and the plasmodesmata in the anticlinal walls of the head cells. (f) Elongated leucoplast devoid of inner membranes with homogeneous stroma surrounded by endoplasmic reticulum. (g) Dictyosomes and vesicles, marked by ZIO reaction, and endoplasmic reticulum profiles close to plasmodesmata (arrowhead). Note the dense materials close to the plasma membrane and in the periplasmic space (arrows). (h) Stalk cell with thin anticlinal walls and thickened lateral walls showing central nucleus and endoplasmic reticulum. (i) Gland foot formed by tangentially expanded cells with dense inclusions in the vacuoles. Note cytoplasmic oil body. Key to figures: cw, cell wall; di, dictyosome; er, endoplasmic reticulum; is, intercellular space; le, leucoplast; mi, mitochondria; nu, nucleus; ol, oil body; pl, plastid; sg, starch grain; sh, secretory head; st, stalk; va, vacuole.

From young floral buds to second-day flowers, ENN were active in secretion. Under TEM, the secretory head cells in young buds were featured with large nuclei with organized nucleoli, dense cytoplasm, poorly developed or absent vacuoles (Fig. 9a), and amyloplasts with abundant starch grains (Fig. 9b and c). ZIO reaction showed that the dictyosomes were well-developed and active in vesicle production (Fig. 9c). In pre-anthesis buds (Fig. 9d–i), the head cells had a lot of mitochondria and long rough endoplasmic reticulum profiles (Fig. 9d). Leucoplasts had residual starch grains and big osmiophilic inclusions (Fig. 9e). Elongated plastids devoid of inner membranes and presenting homogeneous stroma and periplastidial reticulum (Fig. 9f) were found in the head cells. Plasmodesmata were seen in the anticlinal walls connecting the secretory head cells (Fig. 9e and f). Golgi bodies cisternae and vesicles with electron-dense material (Fig. 9g), marked by ZIO, were found in the head cells. Stalk cells had thicker lateral walls and very thin anticlinal walls (Fig. 9h). They also had large nuclei with evident nucleolus, dense cytoplasm with a lot of rough endoplasmic reticulum, and small vacuoles (Fig. 9h). Gland foot cells were tangentially expanded and had small vacuoles with dark inclusions, as well as cytoplasmic oil bodies (Fig. 9i).

**Figure 10.**
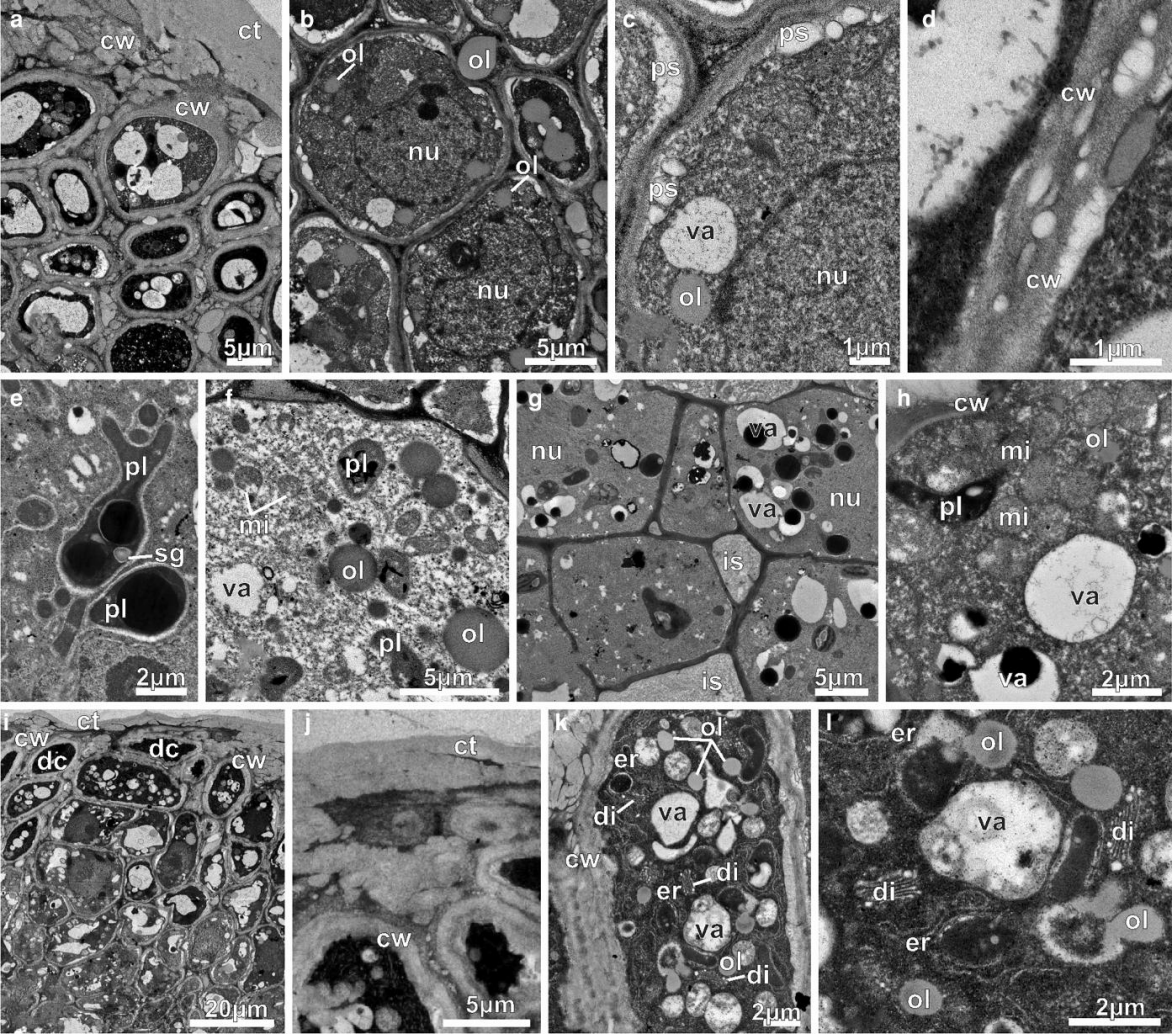
Transmission electron micrographs of the ENN of *A. mansoanum* (Bignoniaceae). (a–h) First-day flowers; (i–l) second-day flowers. (a) General view showing thick cuticle with accumulations of electron dense material. Note the dark and clear cells with thicker walls. (b) Head cells with voluminous nucleus, abundant cytoplasm, and large lipid drops. (c) Periplasmic spaces along the anticlinal walls containing paramural bodies. (d) Lipid inclusions in the anticlinal walls of the head cells. (e) Polymorphic plastids with finely granular stroma and osmiophilic inclusions (likely terpene/phenols). (f) Stalk cell with large lipid bodies, mitochondria, and small vacuoles. (g) Gland foot with wider intercellular spaces filled with fibrillar material, and cells with prominent nucleus, abundant cytoplasm and small vacuoles with electron dense bodies. (h) Detail of a foot cell with numerous mitochondria, electron-dense plastids and small vacuoles. (i) Apical region of the head with abundant dark cells. (j) Larger accumulations of electron-dense material in the cuticle. (k) Dark cells with abundant cytoplasm, rich in intact organelles and small vacuoles. (l) Dark cell showing well-developed Golgi bodies with numerous cisternae, sparse profiles of endoplasmic reticulum, oil bodies, and vacuoles with membrane debris. Key to figures: ct, cuticle; cw, cell wall; di, dictyosome; dc, dark cells; er, endoplasmic reticulum; ie, intercellular space; mi, mitochondria; nu, nucleus; ol, oil drop; pl, plastid; ps, periplasmic space; sg, starch grain; st, stalk; va, vacuole.

In first-day flowers, cuticle was thicker and contained accumulations of lipids and an electron dense material (Fig. 10a). The nuclei were central and spherical, with abundant cytoplasm displaying large lipid droplets (Fig. 10b). At this stage, there were periplasmic spaces and paramural bodies along the anticlinal walls (Fig. 10c), which exhibited areas with lipid inclusions (Fig. 10d). These cells contained polymorphic plastids with finely granular stroma and osmiophilic inclusions (Fig. 10e), likely terpene/phenols. In stalk cells, there were also lipid bodies, mitochondria, and small vacuoles (Fig. 10f). In the gland foot, intercellular spaces increased in size and were filled with flocculent material (Fig. 10g). In foot cells, the vacuoles were small, contained electron-dense bodies (Fig. 10g) and numerous mitochondria (Fig. 10h). In the secretory head of second-day flowers there were more dark cells (Fig. 10i) when compared with the previous stage (Fig. 10a). In addition, large accumulations of electron dense material were visible in the cuticle (Fig. 10i and j). The dark cells contained abundant cytoplasm with intact organelles and small vacuoles (Fig. 10k), globose mitochondria, well-developed Golgi bodies with numerous cisternae, sparse profiles of endoplasmic reticulum, and oil bodies (Fig. 10l).

## Discussion

The location and morphology of the nectary disk (NN) of *A*. *mansoanum* follow the pattern observed in other Bignoniaceae species (Gentry 1974, Elias and Gelband 1976, Galetto 1995, Rivera 2000a, 2000b, Guimarães et al. 2016, Frazão et al. 2020), as do the location and morphology of patelliform-concave glands (ENN), as reported in different Bignoniaceae taxa (Subramanian and Inamdar 1985, Galetto 1995, Rivera 2000b, Díaz-Castelazo 2005, Gama et al. 2013, Nogueira et al. 2013, Fróes et al. 2015). The morphology and anatomy of the annular disks of *A. mansoanum* flowers aligns with the pattern suggested by Nepi (2007), being featured with uniseriate epidermis, nectary parenchyma, and a wider densely vascularized subnectary parenchyma. The patelliform glands in *A*. *mansoanum* flowers have extended margins that project above the calyx surface.

Both nectaries exhibit cellular apparatus typical of nectar secretion; however, NN and ENN display distinct structural characteristics, which likely lead to varied nectar features (volume, concentration, and chemical composition).

Patelliform glands of *A*. *mansoanum* have a complex structure consisting of three specialized regions: non-vascularized foot, stalk, and secretory head. For some authors, such glands represent a symplesiomorphy of the group and are derived from peltate trichomes, showing similar anatomical structure, but different shapes, differential proliferation and cell expansion (Rivera 2000b, Gonzalez 2013, Nogueira et al. 2013). Moreover, their stalk layers are considered an equivalent feature, differentiated by cell number and shape and by the number of cell layers (Fróes et al. 2015, Machado and Rodrigues 2021).

Similarly to other Bignoniaceae species (Guimarães et al. 2016, 2018, Machado et al. 2016), we saw changes in NN and ENN ultrastructure along flower development. These changes likely contribute to the differences in NN and ENN traits of *A. mansoanum* recorded by Balduino et al. (2023).

NN and ENN showed a significant difference in their sugar sources. In NN, starch grains, stored in the parenchyma cells during flower lifetime, are depleted during the pre-anthesis stage and on the first day of anthesis, as reported by Pacini et al. (2003). This information associated with NN vascularization indicates that NN can get their energy directly from the phloem. In turn, starch grains are absent or present in minimal amounts in ENN. Thus, the nectar sugar may originate directly from photosynthesis within the nectary or from the calyx tissues surrounding the gland foot, as described in non-vascularized nectaries (Nepi 2007). It is noticeable that the foot cells of the patelliform glands (immersed in the calyx mesophyll) have more abundant plasmodesmata when compared with other gland regions. The calyx’s vascular tissues end at the base of the gland, and do not penetrate the foot of the gland. Pre-nectar likely exits the sieve tubes through an apoplastic route via intercellular spaces, reach the gland foot, and follow a symplast route in an ascending Trajectory through plasmodesmata, reaching the stalk cells. The apoplasmic barrier in the lateral cell walls of the stalk cells directs the pre-nectar towards the secretory head (Fahn 1979, Machado and Rodrigues 2021). In *A. mansoanum*, there is a substantial difference in nectar volume between the two types of nectaries (Balduino *et al*. 2023). Although this is not a general rule, this pattern is consistent with what has been reported by Díaz-Castelazo et al. (2005) for other ENN, as NN are directly vascularized, while ENN are smaller and non-vascularized. However, the clustered ENN, considered an adaptive trait (Elias and Newcombe 1979), may collectively produce enough nectar to ensure ant visitation to the calyx surface.

It is noticeable that the development level of the phloem and xylem in NN varies between pre-anthesis buds and open flowers, with a predominance of phloem in pre-anthesis buds. During this stage, phloem was characterized by large-calibre sieve tubular elements, indicating that non-reducing sugars (and other solutes) are being unloaded into the subnectary parenchyma where they will be stored as starch grains (Taiz et al. 2013). In most Bignoniaceae species, NN is supplied by phloem elements, except for *Tabebuia heptaphylla* (Vellozo) Toledo, in which they are vascularized by both xylem and phloem (Galetto 1995). A correlation between nectar concentration and NN vascularization was highlighted by Frey-Wysling (1955) but remains poorly investigated. Our study demonstrated that the NN of *A. mansoanum* is vascularized with phloem and xylem. The appearance of tracheary elements in the subnectary parenchyma of first-day flowers indicates a change in the translocation route and pre-nectar components, which is common depending on the stage of organ development and on the vascular connections between source and sink (Taiz et al. 2013). This change means that water, supplied both by phloem and xylem, is being used to compose nectar, which may result in a more diluted secretion with lower sugar concentration. This is consistent with the characteristics of *A. mansoanum* NN reported by Balduino et al. (2023), which was found to be less concentrated and more abundant than that of other Bignonieae species (Galetto 1995, 2009). Both bee pollinator species reported visiting *A. mansoanum* flowers are from the Centridini tribe (Yanagizawa and Gottsberger 1982, Balduino et al. 2023). Although they prefer nectar with higher sugar concentrations (Roubik *et al*. 1995), it is possible that they can use physiological and behavioural adaptations to concentrate diluted nectar (Nicolson 2009), which may allow them to explore a wider range of nectar concentrations (Galetto 2009), as well as different volumes.

The flowers of *A. mansoanum* quickly release a lot of NN (Balduino et al. 2023). This might happen because of the large amyloplasts in the parenchyma cells, which differentiate before secretion and lose most of their starch grains. Thus, starch grains were either used to make nectar sugars or to provide energy for the secretory process (Fahn and Shimony 2001, Stpiczyńska et al. 2005, Heil 2011, Guimarães et al. 2018). Based on a disk surface SEM study, it appears that the modified and raised stomata of *A. mansoanum* primarily facilitate nectar release (Gaffal et al. 1998, Wist and Davis 2006, Tölke et al. 2015). TEM examination of the cuticle that covers the disk epidermis revealed a reticulated structure, which may enhance permeation and facilitate the release of fat-soluble substances (Paiva 2016, 2017). The observation of secretion residues on the cuticle adds to the evidence that lipophilic components of secretion are also being released through microchannels. Ascensão et al. (1997) suggested that microchannels constitute the exit route of volatile substances. On the other hand, secretion accumulates in small subcuticular spaces in patelliform glands, particularly within the central epidermal cells, and is released through the cuticle or small pores. These findings suggest that ENN release occurs continuously, while NN releases secretion all at once through raised stomata. These findings align with the variation of NN and ENN production in space and time. NN has a short lifespan and is active in secretion from pre-anthesis buds to second-day flowers, while ENN is active from young buds to senescent flowers, and apparently to young fruits.

In general, the ultrastructural organization of the epidermal secretory cells in both NN and ENN is made up of a complex system of endomembranes, including the endoplasmic reticulum, dictyosomes, and vesicles. Dictyosomes in the gland cells are associated with the production of hydrophilic substances, while free ribosomes and rough endoplasmic reticulum elements can be related to the synthesis of enzymes involved in starch grain hydrolysis and degradation processes, which were observed during nectar secretion (Paiva and Machado 2008, Possobom et al. 2010, Tölke et al. 2015, Guimarães et al. 2016, 2018). However, the nectaries of *A. mansoanum* differ in abundance and volume of amyloplasts, mitochondria, oil droplets, and degree of rough endoplasmic reticulum (RER) development. Moreover, the presence of fibrillar proteins was observed only in NN, while plastids with periplastidial reticulum were observed only in ENN. Recent findings highlight how mitochondria, along with plastids and the cytosol, help to produce terpenes (Pazouki and Niinemets 2016). Thus, the increase in number of mitochondria observed here can be indicative of their involvement in terpene synthesis in NN. The increase in the presence of lipids is commonly reported throughout nectary development (Tölke et al. 2015, Guimarães et al. 2016, 2018). The presence of oil droplets in vacuoles, scattered throughout the cytosol and within the periplasmic spaces, which are more abundant in ENN than in NN, is ultrastructural evidence of lipidic secretion (Canaveze et al. 2021, de Deus Bento et al. 2024). Plastid morphology in the secretory cells is quite similar to that of plastids involved in the synthesis of terpenes/phenol in lipophilic glands (Kowalkowska et al. 2012, 2015, Machado et al. 2023), but is uncommon in nectariferous tissues (Guimarães et al. 2018). Numerous studies have indicated that plastids are involved in the synthesis of volatile compounds, primarily of phenolic and terpenoid compounds (Pridgeon and Stern 1985, Stern et al. 1987, Pais and Figueiredo 1994, Kowalkowska et al. 2012, Stpiczyńska and Davies 2016, Guimarães et al. 2018, Wiśniewska et al. 2018). The presence of polymorphic plastids containing lipophilic inclusions provides strong evidence that these structures are involved in scent production (Anton et al. 2012, Kowalkowska et al. 2015, Guimarães et al. 2018). Additionally, periplastidial reticulum contributes to the translocation and/or temporary concentration of lipophilic secretions (Sá-Haiad et al. 2015, Canaveze et al. 2021). It is possible that the osmium tetroxide-darkened material in the plastids, cytoplasm, RER, and vacuoles is made up of unsaturated lipids, terpenes, and phenolic compounds (Kowalkowska et al. 2015, Naczk et al. 2018), since osmium tetroxide is known to have better penetration properties and a strong affinity for double bonds (Angermüller and Fahini 1982). This is consistent with our histochemical tests that revealed lipids, terpenes, and phenolic compounds in the secretory cells. Despite the low specificity of the histochemical tests, Sudan IV and NADI reagent have been widely used to, respectively, locate total lipids and terpenes in secretory tissues (da Silva Figueiredo et al. 2007). Lugol’s reagent, containing potassium iodide, can mark alkaloids in plant cells, in addition to staining starch grains (da Silva Figueiredo et al. 2007).

In NN, the presence of polysaccharides throughout the nectary, in cell walls and cytoplasm, keeps the tissue hydrated and may facilitated pre-nectar transport through nectary cells. As in NN, polysaccharides may also be related to nectar pathways in ENN. Their presence in the secretory head may also contribute to the viscosity of nectar (Fahn and Benouaiche 1979), which is a feature that affects ant foraging behaviour (Lois-Milevicich et al. 2021) and may be related to the protection of nectar from desiccation and pathogens. Additionally, although further investigation is needed, polysaccharides could modulate volatile organic compound liberation due to their distinct lipophilic nature. Thus, this could be an ecologically relevant feature, as ants rely on chemical cues for their activities (Knaden and Graham 2016, Nelson et al. 2019).

Both types of nectaries produce hydrophilic and lipophilic secretion at different developmental stages as reported in other bee-pollinated Bignoniaceae species (Baker 1977, Guimarães et al. 2018), which reveals the plastic potential of secretory cells. Some authors associate the presence of lipids to environmental conditions, since they may help prevent water loss and increase nectar’s availability period (Cobert et al. 1979, Stpiczyńska et al. 2021, da Silva et al. 2025). Although this rationale applies to ENN, one can also interpret this result in terms of how valuable lipophilic compounds are for plants, as they can be floral scent compounds related to either attraction or repellence of animals, which may be mutualists or antagonists (Slavković and Bendahmane 2023). For animals, they can constitute part of dietary requirements that enhances their health (Barberis et al. 2023), movement (Gilbert et al. 1965, Levin et al. 2017), development, and reproduction (Stone et al. 1985, Canavoso et al. 2001, Vaudo et al. 2015). Additionally, finding a source of nectar that contains lipids can benefit bees, such as *A. mansoanum* pollinators that collect oil or mix oil with nectar to feed their larvae since lipophilic compounds are more caloric than carbohydrates. Therefore, the presence of lipids and terpenes/phenols in both types of floral nectaries helps explain the diversity of animals with varying needs and ecological roles that visit *A. mansoanum* (Balduino et al. 2023).

Proteins are generally associated with plant defence (Schmitt et al. 2021) and their absence in ENN is a remarkable trait, since the proportion of protein amino acids in ENN is higher than in NN (Balduino et al. 2023). High enzyme activity could explain this result, especially because ENN is more subjected to microorganism arrival (Nepi et al. 2012). The presence of alkaloids in *A. mansoanum* nectary tissues is aligned with its nectar composition (Balduino et al. 2023) and may also protect against pathogens and antagonist herbivores (González-Teuber et al. 2009). Besides, alkaloids also have neuroactive effects on visitors’ foraging behaviour (Barberis et al. 2023, Mogensen et al. 2024).

NN suddenly release nectar right before anthesis starts, so when the flowers open, pollinators can immediately obtain nectar. This implies that the nectar volume available in NN is negatively linked to the number of bee visits received by a given flower, showing the ephemerality of this trophic resource associated to pollination mutualism. In contrast, ENN are active for a longer period of time. They produce nectar daily from the early bud stage until the flower dies, which may take up to 6 days (unpublished data). Even though nectar production stops in the afternoon (Balduino et al. 2023), ENN resume nectar production again early in the morning of the following day. This mechanism of long-lasting nectar production and secretion allows ENN to be explored by visiting insects for several days in a single flower, and even for several weeks, if one considers that there are floral buds in different stages within a given inflorescence.

## Conclusion

In *A. mansoanum*, NN and ENN coexist in the same flower and are exposed to similar environmental and physiological conditions. Both nectaries exhibit cellular apparatus typical of nectar secretion; however, they also display distinct structural characteristics. These differences are especially clear when considering for how long each type of nectary secretes and the different groups of animals that visit each nectary. Thus, the changes in both NN and ENN cellular machinery through flower lifetime reflect the functioning of both nectaries; especially, the duration of their secretory activity and the interactions in which each nectary type is involved. Therefore, understanding how the different nectaries within a flower function can enhance our comprehension of their ecological roles in mediating interactions between plants and animals.

## Data Availability

The data underlying this article are available in the article.
